# Supplementary material for: Non-Invasive Estimation of Central Systolic Blood Pressure by Radial Tonometry: A Simplified Approach
Source: J Pers Med. 2023 Aug 10;13(8):1244. doi: 10.3390/jpm13081244 (PMC10455683; doi:10.3390/jpm13081244)

Supplementary Materials

Non-invasive estimation of central systolic blood pressure by radial tonometry: a simplified approach

from

Denis Chemla [1] , Davide Agnoletti [2, 3]*, Mathieu Jozwiak [4, 5] , Yi Zhang [6, 7], Athanase Protogerou [8], Sandrine Millasseau [9], Jacques Blacher [6].

Supplementary Material ESM1: Rationale for the DCBP formula.

The rationale behind the DCBP is approach is as follows. Firstly, a previous invasive pressure study relying on high-fidelity pressure recordings has demonstrated that the time-averaged central mean blood pressure (cMBP) can be accurately estimated by calculating the geometric mean of central systolic blood pressure (cSBP) and central diastolic blood pressure (cDBP) (13).

$$\mathrm{cMBP} = \sqrt{\mathrm{cSBP\ x\ cDBP}}$$

Consequently, it can be inferred that

$$\mathrm{cMBP}^2 = \mathrm{cSBP} \times \mathrm{cDBP}$$

$$\mathrm{cSBP} = \mathrm{cMBP}^2 / \mathrm{cDBP}$$

It is known that MBP and DBP in peripheral large arteries undergo minimal changes compared to their central counterparts (14-17). This gives rise to the DCBP formula to estimate cSBP solely from peripheral pressures:

$$\mathrm{DCBP} = \mathrm{MBP}^2 / \mathrm{DBP}$$

Table S1: DCBP calculated from the radial MAP estimated by the rule of thumb.

**Variables**	**Mean ± SD**	**Range**
Brachial SBP, mmHg	136.4 ± 18.3	99 - 211
Brachial DBP, mmHg	80.7 ± 10.4	61 - 110
Brachial PP, mmHg	55.1 ± 13.3	33 - 101
Radial MAP (33%), mmHg	99.4 ± 12.0	74.3 – 134.0
cSBP (SphygmoCor), mmHg	123.1 ± 18.3	86.0 – 181.0
DCBP-33%, mmHg	123.9 ± 17.3	89.0 – 196.9
Error, mmHg	0.8 ± 9.0	-14.6 – 34.9
Error, %	1.0 ± 7.3	-11.5 – 26.9

cSBP: central systolic blood pressure. DBP: diastolic BP. DCBP: Direct central Blood Pressure estimation. MAP: mean arterial. PP: pulse pressure. SBP: systolic blood pressure. DCBP was calculated as the radial MAP^2/brachial DBP ratio. DCBP-33% was calculated using the rule of thumb to estimate radial MAP (DBP+33%PP). Error = DCBP – cSBP difference, expressed in mmHg or as a percentage of cSBP.

Figure S1

Receiver-Operating Characteristic analysis.

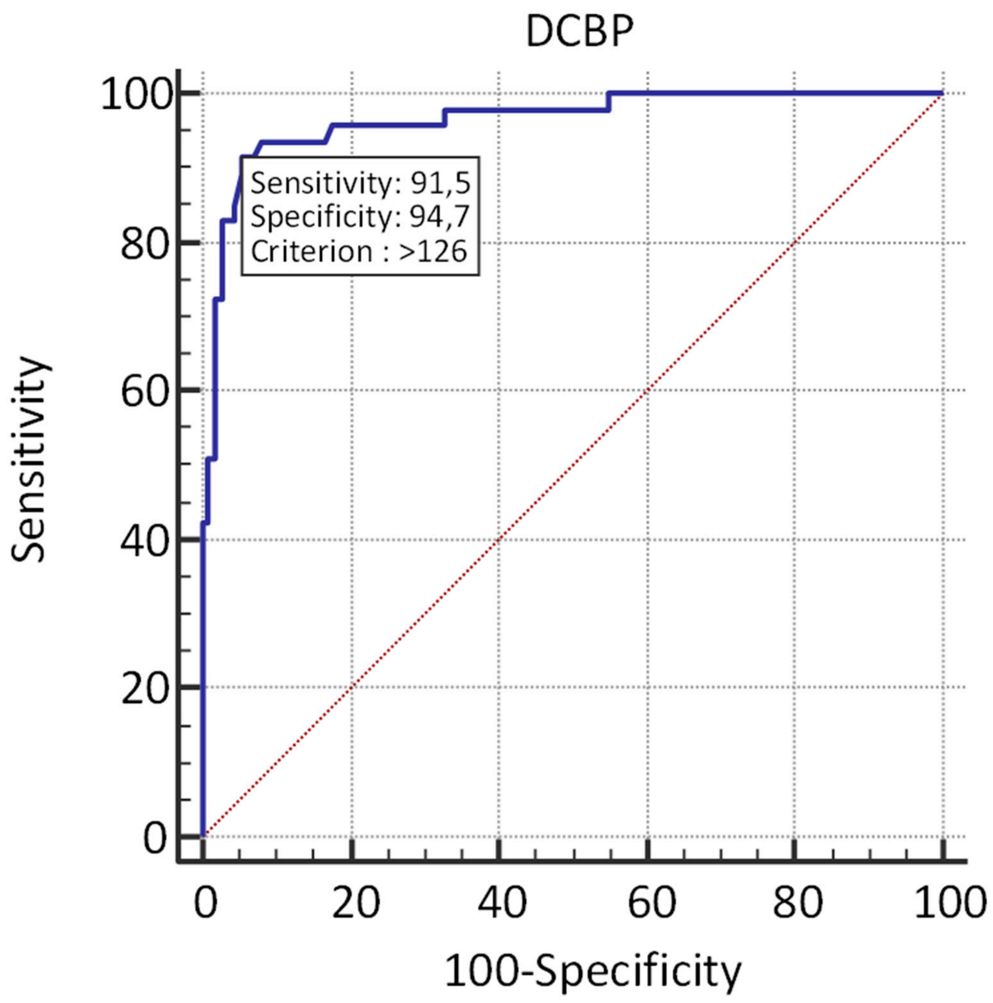

The cSBP was ≥130 mmHg in 47/160 subjects (29%). A DCBP value > 126 mmHg exhibited a sensitivity of 91.5% and specificity of 94.7% in discriminating a cSBP threshold of 130 mmHg (Youden index = 0.86; AUC = 0.965 (95%CI 0.923 to 0.987)).

Figure S2.

Bland & Altman plots with DCBP calculated from the radial MAP estimated by the rule of thumb

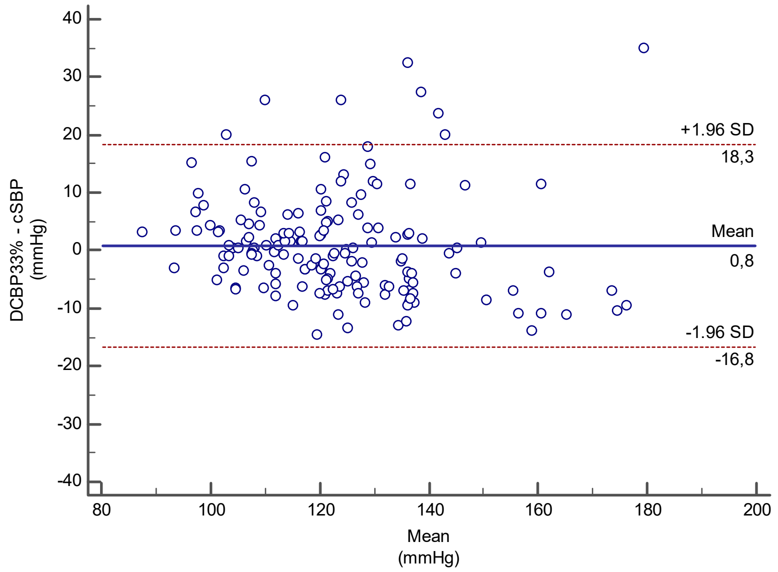

Supplement: Supplementary file 1 [file jpm-13-01244-s001.zip › jpm-2509383-supplementary.pdf]
